# To Explore the Experiences of Women on Reasons in Initiating and Maintaining Breastfeeding in Urban Area of Karachi, Pakistan: An Exploratory Study

**DOI:** 10.5402/2011/514323

**Published:** 2011-05-19

**Authors:** Zahra Shaheen Premani, Zohra Kurji, Yasmin Mithani

**Affiliations:** ^1^University of London, 32 Russell Square, London WC1B 5DN, UK; ^2^Catco Kids, Bahria Complex 2, 1st Floor, MT Khan Road, Karachi, Pakistan; ^3^The Aga Khan University School of Nursing, Karachi 3500, Pakistan

## Abstract

This is an exploratory study that explores the experiences of lactating women in initiating, continuing, or discontinuing breastfeeding in an urban area of Karachi, Pakistan. *Objectives*. To explore the experiences of lactating women and to understand their support and hindering mechanisms in initiating and maintaining breastfeeding. *Methods*. This is an exploratory design assisting in exploring the participant's experiences of initiating and maintaining breastfeeding to better understand their world. Purposive sampling was used, and data was analyzed through manual thematic analysis. *Results*. The data revealed that mother's knowledge, sociocultural environment, breastfeeding decision, and self- and professional support acted as driving forces for the participants. However, sociocultural environment, physiological changes, time management, and being a housewife to breastfeed their children were all challenges and barriers that the participants thought hindered their breastfeeding initiation and maintenance. *Conclusion*. Breastfeeding is a natural but taxing phenomenon, and breastfeeding mothers experience supporting and hindering factors in initiating and maintaining breastfeeding.

## 1. Introduction

Breastfeeding is a natural phenomenon having numerous benefits for both the mother and baby. Breastfeeding is the perfect way to provide the best food, having all the balanced nutritional contents, for a baby's first six months of life. It is a form of nutrition that demarcates no socioeconomic boundaries and provides the same nutritional content to all children all over the world. Breastfeeding is so much more than food alone; breastfeeding protects babies from diarrhea and acute respiratory infections, stimulates their immune systems, and improves response to vaccinations [1–3]. It promotes a child's overall development including cognitive, psychosocial, and emotional development [4]. Breastfeeding creates a special bond between mother and baby and offers unique interaction and stimulation that—along with the balance of protein and energy and micronutrients—helps growth and development and gives a sense of well-being and security. Breastfeeding also benefits the mother's health by helping the uterus to contract soon after delivery, thus reducing chances of prolonged bleeding. It also helps decrease the mother's risk of ovarian and breast cancer [1–3].

On the contrary, there has been extensive evidence on the fact that formula feeding carries risks of additional illness and death, particularly where the levels of infectious diseases are high and where preparation and storage of these substitutes is not carried out properly [5]. In addition, many studies indicate that a nonbreastfed child living in disease-ridden and unhygienic conditions is more likely to die of diarrhea and other diseases related to gastro intestine than breastfed infants. Other health problems include ear infections, allergies, and dental carries [5, 6].

Health status of the infants in entire South East Asia is alarming, and the entire picture of South East Asia in terms of neonatal deaths is critical, Pakistan being one of the countries [7]. According to Horton in the Lancet magazine:

“Of all deaths in children under the age of five years, nearly 40% occur during the first four weeks of life…. Two-thirds of deaths occur in 10 countries (Afghanistan, Bangladesh, China, Democratic Republic of Congo, Ethiopia, India, Nigeria, Pakistan, Indonesia, and the United Republic of Tanzania).” [7].

Health status of infants in Pakistan presents a gloomy picture. The data reveals that Infant Mortality Rate of Pakistan is very high, accounting to 77 per 1000 live births with diarrhea being the leading cause of the death among other causes [8]. Similarly, economic survey of Pakistan 2002-2003 also reveals that Pakistan has one of the worst records in breastfeeding rates within South Asia. Similarly, assessment of the Global Strategy for infant and young child feeding conducted as part of IBFAN Asia Pacific's World Breastfeeding Trends Initiatives (WBT) in South Asian countries in 2005-2006 compiled by Gupta et al. [9] shows that Pakistan is categorized as C category and ranks the fourth among eight South Asian countries scoring 75.5 out of 150. The report card [9] further demonstrates that:

“In Pakistan,…more than 378,000 babies are likely to die during their first year of life. According to new evidence, 22% of all neonatal deaths could be prevented if breastfeeding is initiated within one hour by all mothers (Pediatrics, March 2006). More than 13% of child deaths could be averted in Pakistan, if optimal breastfeeding practices were scaled up to 90%.” [8].

Hence, the picture of Pakistan is a sorry state leading it towards high Infant Mortality Rates. 

Although breastfeeding has been proven to be the perfect and low-cost food for an infant that prevents neonatal deaths, breastfeeding practices are fading away [10]. In Pakistan, most of the couples live in a joint family system and taking care of babies is a collective responsibility [11]. Furthermore, false beliefs and practices influence breastfeeding practices in Pakistan and due to this, many infants are started on bottle feeding as soon as they are one month old [11]. Thus, breastfeeding rates in Pakistan have been found to be very low. Only 16% of women exclusively breastfeed their infants till 4 months of age [11], while only 56% women are able to successfully breastfeed till 24 months [12]. 

Breastfeeding is a full-time and taxing job and is influenced by various factors. Research studies conducted worldwide demonstrate various reasons that hinder breastfeeding practices. A qualitative study conducted by Earle [3] to study the factors affecting breastfeeding initiation indicates that health promotion campaigns in United Kingdom, early infant feeding decisions, desire for paternal involvement, and the desire to reestablish their identities were some of the leading factors that participants thought of as important in initiating breastfeeding. 

The above literature agrees that breastfeeding is important and ideal for the overall growth and development of the healthy infant. It is not only beneficial for infants but it is also useful for the mothers. There are several quantitative studies conducted in Pakistan that reveal that there are many factors which help nursing mothers to initiate or discontinue breast feeding [10, 11, 13]. However, as per our knowledge, there is a wide gap of qualitative studies that explore this phenomenon. Situation in Pakistan shows a sad state, and, therefore, this study intends to explore the perceptions of lactating mothers to get an insight of the reasons that influence breastfeeding practices so that it could be incorporated as a holistic health care program. This study is important in guiding the health care professionals on developing a holistic plan to assist women to initiate breastfeeding early, thereby increasing breastfeeding rates and improving health outcomes.

## 2. Materials and Methods

### 2.1. Study Design

This is an exploratory design that explores the experiences of breastfeeding women.

### 2.2. Sample Size

Six women who are currently breastfeeding or have discontinued breastfeeding between January 2009 to August 2009 with ages 20 to 35 years are selected, out of which 3 are working and 3 are nonworking women.

### 2.3. Sampling Technique

Purposive sampling is used. The participants were selected according to the specific purpose of our study, as per the sampling criteria. This entailed women selecting women who are currently breastfeeding or have discontinued breastfeeding their children. As we are studying the perceptions of breastfeeding women, purposive sampling was most suitable.

### 2.4. Sample Selection

#### 2.4.1. Inclusion Criteria

The inclusion criteria for sample selection consisted of women aged 20–35 years who are currently breastfeeding (exclusive/mixed feeding-breastfeeding along with bottle feeding), women aged 20–35 years who have recently discontinued breastfeeding (during January to August 2009), lactating women who are working as well as who are not working and women who consent to be part of the study.

#### 2.4.2. Exclusion Criteria

Women who were Primiparas (women who are pregnant for the first time), were below 20 years or above 35 years, had discontinued breastfeeding before January 2009, did not have children, or did not consent to be part of the study, were excluded from the study. 

### 2.5. Data Collection Procedure

#### 2.5.1. Methods for Collection of Data

Data is collected via individual in-depth interviews of the six selected participants [14]. Hence, to explore participants experiences on breastfeeding initiation and maintenance, a 45-minute in-depth interview with two separate groups, that is, working women and nonworking women was conducted in the context of the participants. 

Prior to interviews, a letter of permission was sent to the community area from where the women are recruited, along with a personal visit, stating the aims of the study, and requesting an interview schedule before conducting the interviews. Participants were selected on the basis of personal contacts, who meet the selection criteria. Informed consent was taken prior to interview after explaining the form in detail. Prior appointments were taken keeping in mind participant's availability. Refreshments were provided after the interview.

### 2.6. Developing Interview Guide

Keeping in view the research question that would be exploring the experiences of mothers in initiating and maintaining breastfeeding, a semistructured interview guide was developed. This guide was flexible and consisted of open-ended questions for a 45-minute individual in-depth interview (please see Appendix C). Flexibility aided me as the researcher in asking questions as not all individuals perceived the questions in the same way [14]. The interview guide was in English but the interview was conducted in Urdu, as the participants were educated and understood English. The interview guide was pilot tested with a similar participant prior to the in-depth participant interview. Pilot testing assisted me, as a researcher, to get hands on with the interview process and determine any issues with the interview style and/or process. Pilot interview was done with one mother who fulfilled the selection criteria. During the interview, a note taker was present to write the notes. Data was recorded via tape recorders. Research supervisor was involved in the transcription and data analysis process. Bracketing was done to eliminate chances of biasness by writing all my pre-conceived ideas about breastfeeding.

### 2.7. Elements of In-Depth Interview Guide 

Please see Appendix B.

#### 2.7.1. Data Analysis Procedure

Data analysis was simultaneous and done manually as thematic analysis. The field notes and transcription was read many times. The following steps were taken to ensure rigor and check the quality of the data analysis. For instance, codes/pseudonyms were allocated to the participants to maintain anonymity, each transcription and cassette was assigned an ID number, note taker was responsible for recording the data manually and digitally, 2 to 3 days were given for transcription of the data, transcription was done by the note taker, transcription notes were spread to get a complete picture of the participant experiences, analysis was done manually and counterchecked by the supervisor, thematic analysis was done and data was coded, categories and subcategories formed, and themes formulated, and, finally, mentor/supervisor was contacted to check the research process. In addition, digital recorder was used to maintain quality of the data, and spare batteries were kept to avoid technical issues and interruptions in the interviews.

## 3. Ethical Considerations

The proposal and application for approval was sent to the Aga Khan University's Ethical Review Committee (ERC) for the research study. Confidentiality and anonymity was maintained, as codes/pseudonyms are used to maintain the confidentiality of the participant during the dissemination of the results.

## 4. Results

The results of this study demonstrated that breastfeeding mothers experienced various driving and hindering factors that influenced their breastfeeding practices.

### 4.1. Driving Forces/Supporting Factors

**(a) Mother's Knowledge**

The results revealed that mother's own knowledge regarding the importance of breastfeeding played a significant role. All six participants knew the benefits of breastfeeding for both the babies and the mothers and the risks related to bottle feeding.

Data also showed that all six participants were aware of the health hazards and disadvantages of bottle feeding. As one of the participants mentioned:

“It (bottle feeding) is very time consuming, it needs boiling, cleaning and has no benefit. It is very expensive.”

However, regarding benefits of bottle feeding, some of the participants felt that children on bottle feeding are independent and children on breastfeeding are attached with their mothers. Some participants also thought that bottle feeders are chubbier than breast feeders but this did not bother them. 

One of the other participants shared that:

“Initially, I was thinking to introduce bottle feeding (to my baby), but when I took (antenatal) classes, I felt determined for breastfeeding. Although I received feeder bottles as a gift (from other people).”

**(b) Sociocultural Environment**

Sociocultural environment was also considered as an integral support factor in initiating and maintaining breastfeeding. The data demonstrated that family support, husband support, and privacy at workplace were deemed as supporting factors by all 6 participants. Joint family system was seen as a blessing. The participants felt that mother-in-laws' support was very important because this way, the household chores do not suffer. In addition, two of the working mothers felt that the mother-in-law supported in feeding the baby expressed milk. For instance, one participant stated that when she went to attend her classes, her mother-in-law would take care of her child:

“Mother-in-law is supportive. I boil all bottles, keep in fridge and my expressed milk is given (by my mother-in-law) via bottle.”

In relation to husband's support, four of the participants stated that husband support is very important to them. Positive reinforcements by husbands help them continue breastfeeding.

“(It seems like) someone is there to listen. You lie down, I will take care.”

However, 1 participant thought she did not have any support by husband but still she wanted to continue breastfeeding, and 1 participant did not say anything regarding the importance of husband support. Regarding privacy at work, only one participant thought that she did have privacy to feed the baby at her workplace, but still she left her baby home as she was distracted there and thought she could not concentrate on breastfeeding. She mentioned the following:

“Privacy at workplace is provided at my workplace but I feel distracted. There is lack of milk supply because of less fluid intake and not taking Zeera (Cumin seeds).”

**(c) Self**

Results further revealed that all six participants felt that self-motivation of lactating mothers was considered as very important. One of the participants has termed it as “will power.” For example:

“Woman's will power is very important. Because in the beginning, nipples get hard and sitting with baby for long time requires will power otherwise we have to go through a lot of pains “mugusmari karna.” Breastfeeding is a better option.”

One of the other participants highlighted:

“Motivation (of breastfeeding mothers) is 100% important. Mothers should breastfeed….”

According to the other participant,

“Motivation (of breastfeeding mothers) is very important. Internal motivation is necessary. I can become failure. Inner power target is very important.”

**(d) Breastfeeding Decision**

Analysis of data showed that all six participants had decided to breastfeed their babies during pregnancy or before that. Two participants had taken antenatal classes, and two of them were nurses. Experiences of other family members also helped some of the participants. For instance, one of the participants said:

“In my own family, a baby was 2 months old (on bottle feeding) and was suffering from jaundice, stomach ache. I saw it myself…2 months old baby was suffering from constipation.”

Participants who thought antenatal classes and family members helped them make their decision regarding breastfeeding mentioned that:

“My mother-in-law, sister-in-law, doctors, banners at a private hospital motivated me and antenatal class helped me. I decided to breastfeed before pregnancy. I love to breastfeed. I and my child are both satisfied.”

One of the other participants stated that:

“Best time to decide is during pregnancy but when I delivered, I was tired and had many problems. My baby did not know that I had pain but I managed.”

**(e) Professional Support after Delivery**

The data demonstrated that 3 participants experienced that assistance from professional staff had helped them initiate breastfeeding after delivery. They felt it helped them to latch on one of the participants who had her baby after 14 years described that:

“After 14 years, I had my third child. When my first baby was born, nurse at a private hospital motivated me to always breastfeed.”

Another participant even praised the nurses by saying that:

“Nursery staffs are very expert. Although I had the knowledge but experience of the hospital staff was very helpful.”

### 4.2. Hindering Factors/Barriers

**(a) Sociocultural Environment**

Sociocultural environment was viewed as a major hindering factor. All the six participants experienced that cultural norms of the family and the society were barriers towards maintaining breastfeeding. Some family members thought that the child was not gaining weight because of the breastfeeding and that bottle feeders are chubbier. Some of the participant also experienced lack of privacy to breastfeed at home due to joint family system. Hence, extended family was viewed as a challenge by three participants out of six. One of the other participants' family perceived that as the mother was not around the baby most of the day, the baby did not recognize her. For instance, the participant illustrated:

“Those (children) who are bottle fed look healthier and chubby therefore, family pressurized and commented that we should try bottle feeding. But I have observed those who are on bottle feeding they have to rush to emergency ward of a private hospital but Masha Allah, I have never taken my children to Emergency Room.”

Five participants thought that breast feeding in public was a major barrier. All of the participants said that they continued with breastfeeding. As one of the participants verbalized,

“It is (culture) a big challenge. I breastfeed in weddings and in marriage halls. My child is my priority.”

Finally, one of the participants felt very strongly that:

“Mother should feel proud. We should not care about society. I never hesitate (breastfeeding). We should first think of our baby and not of the society.”

**(b) Physiological Changes**

In terms of physiological changes after delivery, all six participants reported that they experienced lack of sleep and rest due to breastfeeding. However, some participants reported that they had breast tenderness, lack of fluid intake, tiredness, backache and child bonding. As one of the participants expressed,

“(I had) lack of sleep. I was not able to take plenty of rest. Major issue was rest but now, habit is developed. I still want to feed.”

One of the other participants mentioned that:

“Till 2 years, (I experienced) lack of sleep. In the night, especially, I have to wake up but now, only 3 months are left. I have never thought of bottle feeding.”

The other participant thought that:

“(It is) very tough. Water intake was not possible. I felt tired and weak. Continuous breastfeeding was very difficult. Feeding in lying position is not encouraged and this is why feeding in sitting position.”

Likewise, the other participant stated that:

“Time management with work (household chores), child crying and especially feeding at night (is challenging). I felt tired, had backache and delivery problems. For the first child, every time we have to get ready for feeding and make sure that (baby's) nose is not pinched and have to be very careful to wake up in the night and feed.”

**(c) Time Management**

Regarding managing time, three participants out of six reported that it is challenging for them to manage time and breastfeeding both. Interestingly, one participant was a housewife and the other two are working women. As the housewife stated:

“…Your entire focus is there (on the child) and you have to do other tasks like cooking, cleaning. So it makes you frustrating.”

The other participant who is currently studying highlighted that:

“The reason for switching (to mixed feeding) is lack of time to express feed. My priority was studying and it was causing many distractions.”

**(d) Role of Housewife**

Analysis of the data revealed that only one participant experienced frustration because of sitting at home after her studies. As she mentioned that:

“During MBA, I left everything and sat at home, so it is very frustrating. I feel angry sometimes for a few seconds and think of discontinuing breastfeeding but you cannot leave breastfeeding.”

## 5. Discussions

**Figure 1:**
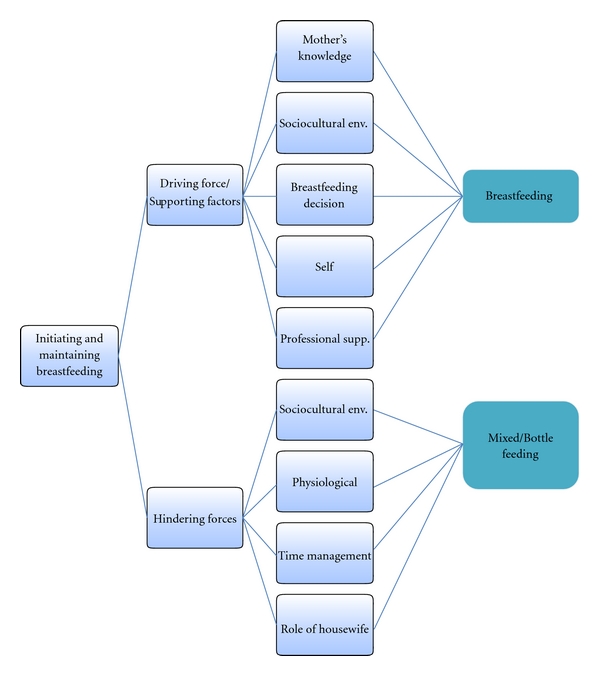


The study conclusion was derived from the participants' data. Various themes were drawn based on the research question and aims of the study. The data revealed that breastfeeding women, both working and nonworking, experienced various factors that supported them as well as acted as barriers in initiating and maintaining breastfeeding. As also suggested by various studies [1, 3, 6, 10, 11, 13, 15–26], mother's knowledge, sociocultural environment like mother-in-law's support, breastfeeding decision before delivery, self-motivation, and professional support acted as driving forces for the participants. However, sociocultural environment such as lack of privacy due to joint family system, physiological changes, time management, and being a housewife to breastfeed their children were all challenges and barriers that the participants thought hindered their breastfeeding initiation and maintenance. As illustrated in Figure 1, if the women are provided with strong support mechanisms, they are likely to breastfeed for a longer duration [27], and if the hindering factors are overwhelming and are not overcome, the mothers may discontinue breastfeeding or resort to mixed feeding method.

Interestingly, sociocultural environment came out to be a supporting factor as well as a hindering factor for the participants. It was seen as a supporting factor by all six participants in terms of family support especially support from the mother-in-law such as support in household chores and feeding the baby when the mother is at work, husband support, for instance, doing other chores while the wife is feeding or burping the baby, and privacy at workplace. Surprisingly, sociocultural factor was also perceived by all six participants as a barrier. The key sociocultural barriers that the participants identified were cultural and societal norms such as bottle-fed babies being chubbier than breastfed babies and lack of privacy to breastfeed in the public as well as at home (due to joint family system). Most of the participants' overcame this challenge by avoiding going to marriages and shopping centers. However, none of them discontinued breastfeeding or opted for mixed feeding because of this reason. 

Participants also expressed that the two supporting factors that assisted them in initiating breastfeeding were their own knowledge and breastfeeding decision prior to delivery of the child. All six participants were aware of the basic advantages of breastfeeding and disadvantages of bottle feeding. According to them, this knowledge helped them overcome family pressures such as “bottle feeders are chubbier.” This knowledge was gained through their own education, antenatal classes, elder family members, media, internet, books, and experience of bottle feeding babies in the family. The literature also demonstrates that mother's knowledge is a strong predictor of breastfeeding [1].

Regarding breastfeeding decision during pregnancy, all six participants highlighted that antenatal classes, motivation, and support from family members facilitated them to initiate breastfeeding. In relation to support after delivery, three participants experienced that professional support from nurses helped them latch on and motivated them to initiate breastfeeding. 

All of the participants also valued self-motivation as an important breastfeeding supporter. They verbalized that if women are motivated, they can overcome various challenges and barriers and will be able to continue breastfeeding. Research studies also indicate that self-motivation [22], prenatal discussions with parents [1] and support from healthcare providers assists in successful initiation of breastfeeding [24]. Various studies also reiterate that mother's attitude, personal beliefs, and conviction have a strong influence on their breastfeeding decision [26].

One of the barriers that was expressed by all six participants as challenging was physiological changes after delivery, which included breast tenderness, lack of sleep and rest, less fluid intake, tiredness, backache, and attachment of the child. This was a cause for frustration for most of them. However, all of them stated that family support (mother-in-law, husband) and professional support played a major role in overcoming this issue. The literature also suggests that physical discomfort is a barrier to breastfeeding [11]. Hence, professional support during this period becomes extremely crucial in continuing breastfeeding. Researches show that breastfeeding mothers who received professional support breastfed longer than those who did not [1]. Some of the participants even mentioned that they thought of discontinuing breastfeeding due to the frustration; yet, none of the participants actually discontinued breastfeeding because of it. 

Time management was also seen as a hindering factor by 3 participants in maintaining breastfeeding. Out of the 3 participants, 1 was a housewife and 2 were working mothers. The participants experienced that lack of time was as a result of household chores and lack of time to express breastfeed. One of the participants also pointed out that she opted for mixed feeding as a consequence of lack of time she had to express her feed.

One of the interesting results that showed up was that only 1 participant voiced that her role of being a housewife during her studies was a challenge for her. She expressed that sometimes when she had to feed the baby and take care of the house, she would become frustrated for a while and thought of discontinuing breastfeeding. Nevertheless, she was intrinsically motivated and, hence, overcame this challenge. 

The results of this study can aid in influencing and increasing breastfeeding rates whereby improving health outcomes. Most importantly, as pioneer Lactation Consultants in Pakistan, the findings of this study will help initiate breastfeeding clinics and design context- and need-based Lactation Consultant certification modules in our country thereby increasing the pool of Lactation Consultants. It will also assist Lactation Consultants in understanding the problems of lactating mothers and in providing empathetic and appropriate counseling.

## 6. Limitations

Time constraint was found to be one of the major limitations of the study due to which triangulation of data collection method was not used and only in-depth interview was used. Though qualitative research studies do not require large sample sizes as they do not intend to generalize the findings, however, due to time constraints, only 6 participants were selected for the study and saturation of the data was not possible.

In addition, being a novice researcher, it was the first time for me to conduct in-depth interviews, manual analysis, and so forth. This was overcome by getting guidance from the research study supervisor and discussing all the research steps before implementation. Study setting was limited to only one for working group and one for the nonworking group.

## 7. Conclusion and Recommendations

In short, all six participants viewed breastfeeding as important for the child. All of them found that support systems are very crucial in initiating and maintaining breastfeeding. All of them also experienced various challenges, which they resolved through the support system that were available for them as well as self-motivation. However, only one participant was practicing mixed feeding due to her busy study schedule and lack of time to express milk. They conveyed their message that anyone who can breastfeed should do so unless there are any issues. One of the participants also verbalized that the decision to discontinue breastfeeding should not be abrupt even if there is a problem. As one of the participants described that,

“Breastfeeding is very important. This is every child's right. “Ye sab bacchhon ka haq hai”. Every child should get it. It is very important for mother's health also and the child is prevented from many problems….”

Thus, the recommendations that have been outlined keeping in view the above findings. As there is a dearth of local qualitative researches, it becomes imperative to conduct further studies to explore parents' perceptions of different aspects of breastfeeding like father's role. Therefore, further qualitative research needs to be done in this area. As it was revealed in this study that husband's role was considered as important in maintaining breastfeeding, it could be further explored. Moreover, as one of the participants verbalized that staying at home caused frustration, this could be further explored that what the other reasons that she opted to stay at home were and she look for other support systems outside her home, for example, quality daycare centers. Prenatal classes and access to information came out to be a strong motivator for breastfeeding decisions and should, thus, be promoted. Public environments that promote breastfeeding should be made conducive so that breastfeeding working women can comfortably breastfeed their children in public too. Workplace support that would include protected time for expressing breast milk or feeding babies excluding lunch hours, paid maternity and paternity leaves, and so forth, would assist in supporting breastfeeding mothers. Lastly, personal support from family and husbands would also encourage mothers towards optimum breastfeeding. 

In conclusion, this small scale study concluded that there are several supporting and hindering factors that influence breastfeeding practices, and optimal breastfeeding can be promoted by maximizing the supporting factors and minimizing the hindering factors.

##  Summary Statement

Although breastfeeding has been proven to be the perfect and low-cost food for an infant, breastfeeding practices are fading away. Research studies conducted worldwide demonstrate various reasons that hinder breastfeeding practices. Situation of Pakistan shows a sad state, and, therefore, this study intends to explore the perceptions of lactating mothers to get an insight of the reasons that influence breastfeeding practices so that it could be incorporated as a holistic health care program.

##  Precis

Breastfeeding is a natural but taxing phenomenon, and breastfeeding mothers experience supporting and hindering factors in initiating and maintaining breastfeeding.

## Appendices

### A. Informed Consent Form

Introduction I am Ms. Zahra Shaheen from the Aga Khan University Human Development Program studying Early Childhood Development Course 3 and doing a research on exploring perceptions of women on breastfeeding. I want to explore what the experiences of women who are breastfeeding their infants are. Since you are currently breastfeeding or having recently discontinued breastfeeding your infant, I would like to invite you to join this research study.

Background Information Breastfeeding is a natural phenomenon having numerous benefits for both the mother and baby. There has been extensive evidence on the fact that formula feeding carries risks of additional illness and poor health outcomes. However, more than half of all infants are still not exclusively breastfed for the first six months of life, and discontinuation of breastfeeding is still widespread in Pakistan.

Purpose of This Research Study Purpose of the study is to explore the perceptions of mothers who are breastfeeding their infants to get an insight of their experiences regarding breastfeeding.

Procedures In this study, we will ask few questions about your experiences of breastfeeding. An interview of approximately 45 minutes will be held at a convenient place and time. You will be contacted twice or thrice after the interview if there are any queries from the interview as well as to recheck and confirm the data (whatever had been said is written correctly). The interview will be tape recorded and a note taker will be present to jot down the notes from the interview.

Possible Risks or Benefits There is no risk or stress involved in this study except for your valuable time. There is no direct benefit to you also. However, the results of the study may help us to understand better about the reasons behind discontinuation of breastfeeding, and, hence, it will assist us in developing comprehensive healthcare plans that will help breastfeeding women facing the challenges to overcome them and may assist them in continuing breastfeeding. Refreshments will be provided after the interview.

Right of Refusal to Participate and Withdrawal You are free to choose to participate in the study. You may refuse to participate without any harm done. You may also withdraw any time from the study. You may also refuse to answer some or all the questions if you do not feel comfortable with those questions.

Confidentiality The information provided by you will remain confidential. Nobody except principal investigator will have an access to it. Your name and identity will also not be disclosed at any time. However, the data may be seen by Ethical Review Committee and may be published in journal and elsewhere without giving your name or disclosing your identity.

Available Sources of Information If you have any further questions you may contact Principal Investigator (Ms. Zahra Shaheen), on the following phone number 0321-8252693.

Authorization I have read and understand this consent form, and I volunteer to participate in this research study. I understand that I will receive a copy of this form. I voluntarily choose to participate, but I understand that my consent does not take away any legal rights in the case of negligence or other legal fault of anyone who is involved in this study. I further understand that nothing in this consent form is intended to replace any applicable federal, state, or local laws.

Participant's name (printed or typed):
Date:
Participant's signature:
Date:
Principal investigator's signature:
Date:
Signature of person obtaining consent:
Date:

### B. Elements of Interview Guide

Demographic data: Participant's profile

- Name:
- Age:
- Education:
- Profession:
- Number of children and their individual health status:
- Mode of delivery:
- Exclusive breastfeeding/supplemental feeding/weaning diet:
- Number of months/years currently breastfeeding:
- How many children have been breastfed:
- Type of family (nuclear/extended):
- Working/nonworking:
- Working hours:
- What support systems are available for leaving the child when going to work?

Questions in the semistructured interview guide will be based on the following themes.

- Experiences of working/nonworking women in initiating and maintaining breastfeeding.
- Support systems available to initiate and continue breastfeeding.
- Barriers affecting women in continuing breastfeeding.
- Challenges faced by the lactating mothers in initiating and maintaining breastfeeding and how they were overcome.

### C. Individual In-Depth Interview Study Guide

Demographic Data: Participant's profile

- Name:
- Age:
- Education:
- Socioeconomic status:
- Profession:
- Number of children:
- Children's individual health status:
- Mode of delivery:
- Place of delivery:
- Exclusive breastfeeding/supplemental feeding/weaning diet:
- Number of months/years currently breastfeeding:
- How many children have been breastfed (exclusive/mixed):
- Type of family (nuclear/extended):
- Working/nonworking:
- Working hours:
- What support systems are available for leaving the child when going to work?

Q.1 Breastfeeding is known to be the best method for infant feeding. What do you think?
Q.2 Bottle feeding is known to have many health risks for babies like diarrhea. What do you think?
Q.3 Can you tell me something about exclusive breastfeeding?
Q.4 What are your perceptions about breastfeeding?
Q.5 How are you feeding the baby?
Q.6 When did you decide to breastfeed?
Q.7 Why did you choose breastfeeding?
Q.8 How many children have you breastfed?
Q.9 What support did you receive when you initiated breastfeeding?
Q.10 What were the barriers/challenges in initiating and continuing breastfeeding?
Q.11 How did you overcome them?
Q.12 Do you think you were able to initiate breastfeeding due to your motivation?
Q.13 In your view, what were the motivating factors that lead you to breastfeeding?
Q.14 What were the reasons that lead you to choose mixed feeding or discontinue breastfeeding?
Q.15 In your opinion, what are the consequences of early cessation of breastfeeding?
